# Scuttle fly *Megaselia scalaris* (Loew) (Diptera: Phoridae) endoparasitoid as a novel biocontrol agent against adult American cockroaches (*Periplaneta americana*)

**DOI:** 10.1038/s41598-024-59547-w

**Published:** 2024-04-29

**Authors:** Esraa A. Arafat, Lamia M. El-Samad, Mohamed A. Hassan

**Affiliations:** 1 Department of Zoology, Faculty of Science, Alexandria University, Alexandria, Egypt; 2 Protein Research Department, Genetic Engineering and Biotechnology Research Institute (GEBRI), City of Scientific Research and Technological Applications (SRTA-City), New Borg El-Arab City, 21934 Alexandria Egypt

**Keywords:** American cockroach, *Periplaneta americana*, Scuttle fly *Megaselia scalaris*, Biological control, Scanning electron microscope, Zombification behavior, Entomology, Scanning electron microscopy

## Abstract

The American cockroach, *Periplaneta americana* (Linnaeus, 1758) (Blattodea: Blattidae), is one of the most common pests that thrive in diverse environments and carries various pathogens, causing critical threats to public health and the ecosystem. We thus report in this study the first observation of decapitated American cockroaches as a result of infestation with scuttle fly parasitoids. Interestingly, behavioral alterations in the form of zombification-like behavior could be observed in cockroaches reared in the laboratory before being decapitated, implying that the insect targets cockroach heads. To identify this parasitoid, cockroaches’ corpora were isolated in jars, and apodous larvae were observed. Larvae developed into small coarctate pupae, and adults emerged. The scuttle flies were collected and exhibited tiny black, brown, to yellowish bodies. The fly was initially identified based on its morphological properties as a member of the order Diptera, family Phoridae. To provide further insights into the morphological attributes of the phorid species, the fly was examined using a scanning electron microscope (SEM) and then identified as *Megaselia scalaris* accordingly. SEM analysis revealed the distinctive structure of *M. scalaris* concerning the head, mouth parts, and legs. Specifically, the mouth parts include the labrum, labellum, rostrum, and maxillary palps. Although further investigations are still required to understand the complicated relationships between *M. scalaris* and American cockroaches, our findings provide a prominent step in the control of American cockroaches using *M. scalaris* as an efficient biological control agent.

## Introduction

The American cockroach, *Periplaneta americana*, is one of the oldest, most adapted, and largest insects in the world^1–4^. Its distinct emergence and invasive nature in both housing and industrial areas make it a critical pest among known pests. It belongs to the order Blattodea (Blattaria), family Blattidae, which is the most known cosmopolitan peridomestic insect pest^5–7^. It can flourish in different environments, which entails controlling and hindering its infestations to safeguard and maintain the environment^6,7^. It has been demonstrated that exposure to *P. americana* induces cockroach allergens, which may cause asthma during childhood in addition to various symptoms concerning psychological stress^7^. However, the most serious health concerns of *P. americana* are related to its role as mechanical vectors for etiological agents, which are mostly drug-resistant^2^. It is not only defined as one of six cockroach species with about forty-two bacterial species but also shows the highest rates of bacterial contamination in general. Furthermore, it facilitates the spread of pathogenic fungi and some human intestinal parasites through the nosocomial infectious route, food contamination, secretions on various surfaces, and feces^6^. Moreover, external contaminants in the American cockroach cuticle, which transmit the eggs of *Giardia lamblia* (Diplomonadida: Hexamitidae), hookworm (Rhabditida: Ancylostomatidae), and *Ascaris* (Ascaridida: Ascarididae) in addition to *Salmonella* sp. and *Shigella* sp. (Enterobacterales: Enterobacteriaceae), *Pseudomonas aeruginosa* (Pseudomonadales: Pseudomonadaceae), and Proteobacteria (Pseudomonadota) are much more hazardous to human health than internal ones^2,6^. Therefore, exploring new biological control agents is necessary to preclude the spread of this worldwide pest^8^.

Among the biocontrol insects, the genus *Megaselia* (Diptera: Phoridae) has drawn marked interest as a potential biocontrol agent against various insect pest species^9^. It has been identified that there are about 230 genera and 4000 species of scuttle flies in the family Phoridae^9,10^. Family Phoridae is one of the most numerous families of the order Diptera, with diverse habitats and a fairly high number of species, mostly in the genus *Megaselia* with about 1700 defined species^10^.

The humped-back appearance and the costal veins, which extend only to the middle of the anterior wing margins, are a leading characteristic of those flies. Additionally, they have tiny black, brown, or yellowish bodies ranging from 1.0 to 5.5 mm for most species, dim eyes, and a globe-shaped third antennal segment^9,11^. Members of the family Phoridae are significant necrophagous flies exploited as forensic evidence, particularly in enclosed environments^12^. It is difficult to access other necrophagous species under these conditions, making them the predominant source for estimating the minimum postmortem interval^12,13^. Besides, they are used as an indicator of environmental hygiene due to their synanthropic nature^9^.

Flies of the genus *Megaselia* have a wide range of synanthropic feeding habits and a sophisticated distribution^14^. They feed on a variety of animals and plants that decompose organic matter, while their larvae lead predatory and parasitic lives^14^. Additionally, they have substantial medical importance, as reported cases of accidental human myiasis have been reported^12,14^. Well-known infestation targets for these flies are laboratory arthropod cultures, although their tiny size is due to their outstanding capability to penetrate tightly closed containers^15–17^. Thus, flies of the genus *Megaselia* are of notable forensic importance in sealed locations^15^. Among the genus *Megaselia*, *M. scalaris* is a synanthropic scuttle fly species that has a wide and pivotal role in the environment in relation to both medical and economic impacts. In addition, they play a substantial role in forensic investigations^12,14^. They show remarkable success due to their rapid development properties^18^. Importantly, they can produce different types of myiasis, including intestinal, urinary, ocular, pulmonary, vaginal, and cutaneous myiasis. Additionally, they can contaminate chicken eggs by feeding their larvae on cracked eggs^12^. Besides, *M. scalaris* could contaminate colonies in forensic and entomological labs. Furthermore, *M. scalaris* can be utilized as an experimental model species^18^. Furthermore, *M. scalaris* previously reported as parasitoid of the desert scorpion *Mesobuthus eupeus* (Scorpiones: Buthidae)^17^, mantids *Parastagmatoptera tessellate* (Mantodea: Saussure)^14^, honey bees *Apis mellifera* (Hymenoptera: Apidae)^19^, soldiers of the termite *Macrotermes gilvus* (Blattodea: Termitidae)^20^, the southern green stink bug *Nezara viridula* (Hemiptera: Pentatomidae)^21^, and the fall armyworm *Spodoptera frugiperda* (Lepidoptera: Noctuidae)^22,23^. Previous reports showed that *M. scalaris* my attack cockroach nymphs^14,18^. Herein, we report, for the first time, decapitated American cockroaches infested with *M. scalaris*. The insects targeted the cockroach's head to complete their life cycle. The insects were obtained and identified based on their morphological features, providing valuable attributes about their external structure. This is the first study to introduce such delicate details about *M. scalaris.*

## Materials and methods

### Culturing of American cockroach (*P. americana*)

Cockroaches, *P. americana*, were cultured in the Entomology Laboratory, Faculty of Science, Alexandria, Egypt. They were reared under sterile conditions and supplied with sterilized dry food and water with daily cleaning to avoid unanticipated contamination, which might affect the culture.

### American cockroach behavior

Alterations in cockroaches' behavior were observed daily for three weeks. The observations focused on various characteristics, including movement patterns, social interactions, feeding routines, and resting behavior. After two weeks of observation, the cockroaches' behaviors remarkably changed due to an infection that caused their decapitation after three days.

### Parasitoid collection

To find out the reason for cockroach infection, decapitated cockroaches were carefully collected in jars before being tightly sealed with a cloth and maintained at room temperature. After three weeks, larvae hatched in the jars and completed their lifecycle by entering the pupation. Afterward, the pupae developed into adult flies in one week, which were euthanized by placing them in a freezer for 10 min, followed by preservation in 70% ethanol for morphological identification.

### Characterization and identification of parasitoids using SEM analysis

To survey the microstructure of adult flies, the samples were prepared as previously reported^24,25^ with slight adaptations. Briefly, the specimens were euthanized, washed with a normal saline solution several times, and then preserved in 70% ethanol for 24 h before being dried using a critical point dryer (Minnesota, USA). Following this, the specimens were mounted on aluminum stubs and then coated with gold–palladium in a sputter-coating unit (JFC-1100 E). Afterward, the samples were scanned and visualized employing an SEM (Jeol JSM-5300, Tokyo, Japan) at an accelerating voltage of 20 kV. External genitalia, legs, wings, mouthparts, and antennae were examined to define the microstructure properties of this parasitoid. The genus of the fly was identified following Disney, H.^26^, while the species was identified following a dichotomous key. Furthermore, the identification was further corroborated based on their morphological features with the help of Prof. R.H.L. Disney (Department of Zoology, University of Cambridge, United Kingdom).

## Results

### Parasitoid rearing

**Figure 1:**
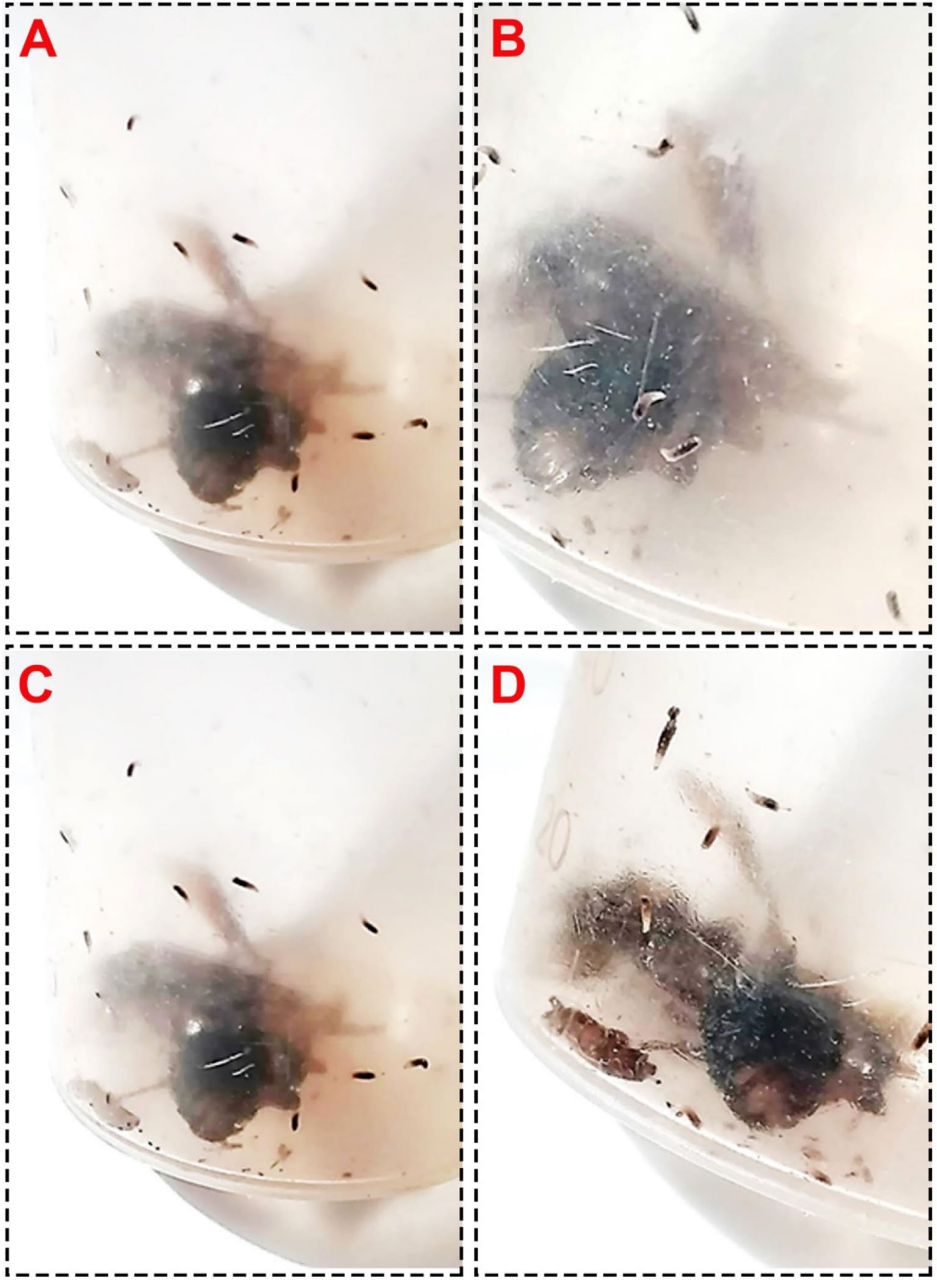
(**A–D**) Photographs of yellowish-black larvae of *M. scalaris* reared from dead cockroaches corpuses in jars.

**Figure 2:**
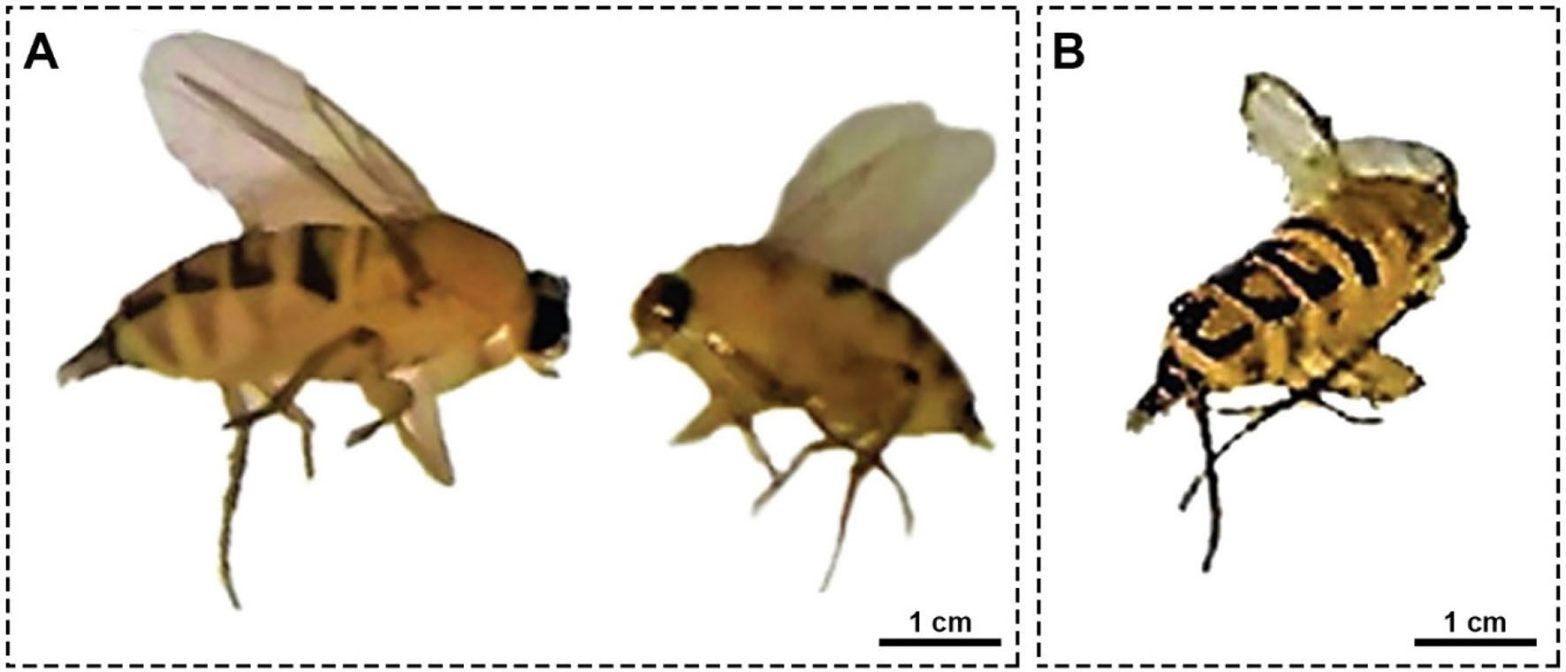
(**A**) Adult female *M. scalaris* is on the left side and male *M. scalaris* is on the right side. (**B**) abdominal tergites of the *M. scalaris* female.

We observed yellowish-black larvae reared from the decapitated cockroach wandering inside the jars as delineated in Figs. 1A–D. After two days, yellow pupae could be discernible, which required five days afterward to develop into adult flies as shown in Fig. 2A and B. The emerged adults were identified as *M. scalaris* on the basis of morphological features. Precisely, it could be perceived from Fig. 2B that flies possess only one pair of wings, while the second one modified to halteres, and a humpbacked appearance with blackish-yellow tergites along their abdomen. Accordingly, the parasitoid was defined as a fly of the order Diptera, family Phoridae.

### Morphological attributes of *M. scalaris* using SEM

**Figure 3:**
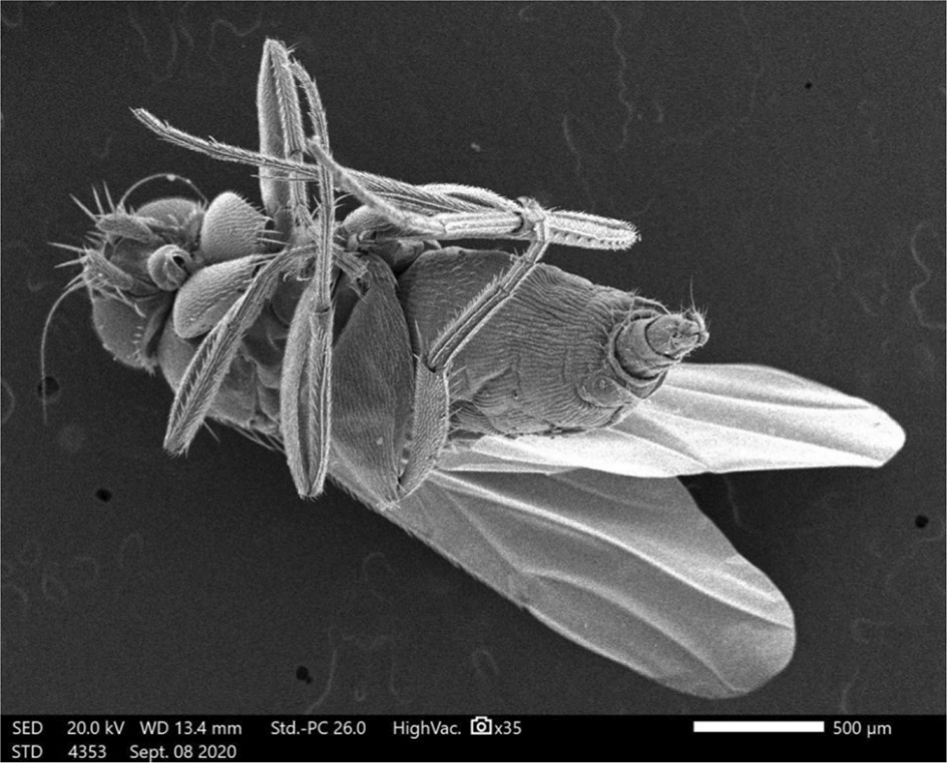
SEM micrograph of the adult female *M. scalaris* showing the ventral view of the whole fly body.

**Figure 4:**
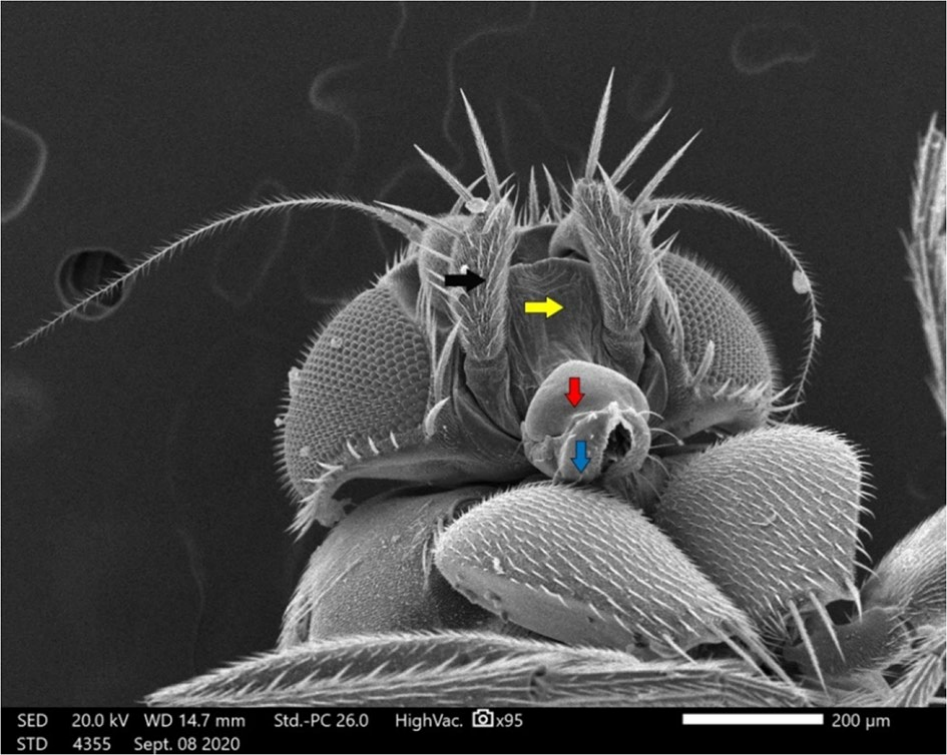
SEM micrograph of the mouthpart structures of female *M. scalaris* illustrates the labrum (red arrow), labellum (blue arrow), rostrum (white arrow), and maxillary palp (dark arrow).

**Figure 5:**
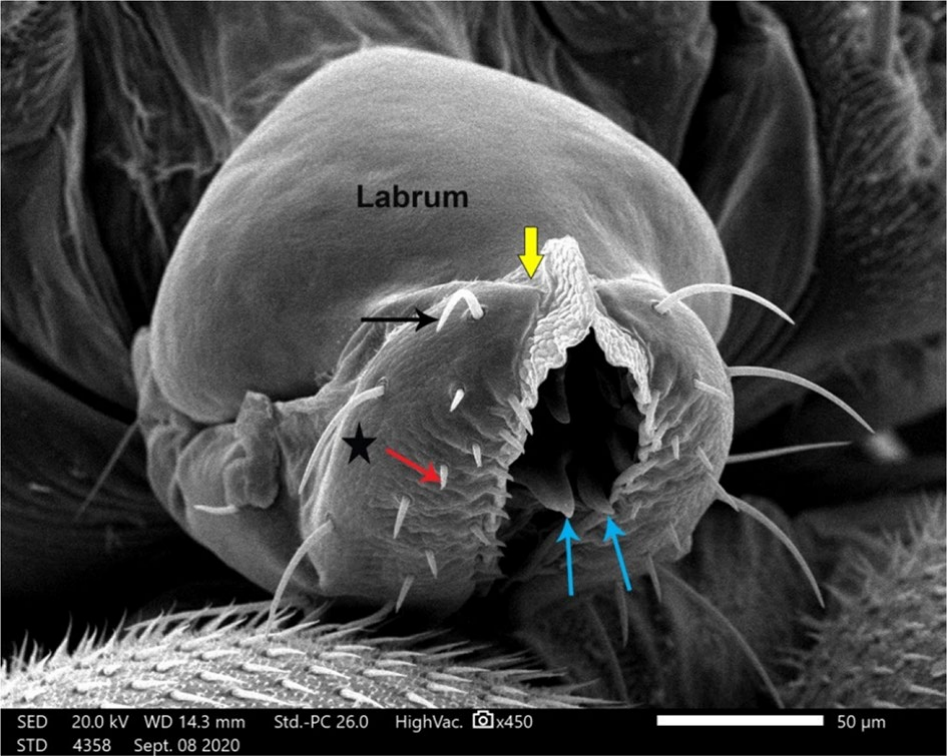
SEM micrograph of the mouthpart structures of the female *M. scalaris*. The ventral view at higher magnification shows the medius membranous lobe (yellow arrow), long trichoid sensilla in a lateral position (black arrow), short in the ventral position (red arrow), the smooth labellum surface without microtrichia (star), and hooks in the food channel (blue arrows).

In our SEM investigation, we focused on the adult female *M. scalaris* structure, which is illustrated by the SEM photomicrographs in Fig. 3. At higher magnification, the chief components of the adult female sponging mouthparts are clear, which are represented in the labrum, labellum, rostrum, and maxillary palps as depicted in Figs. 4 and 5.

**Figure 6:**
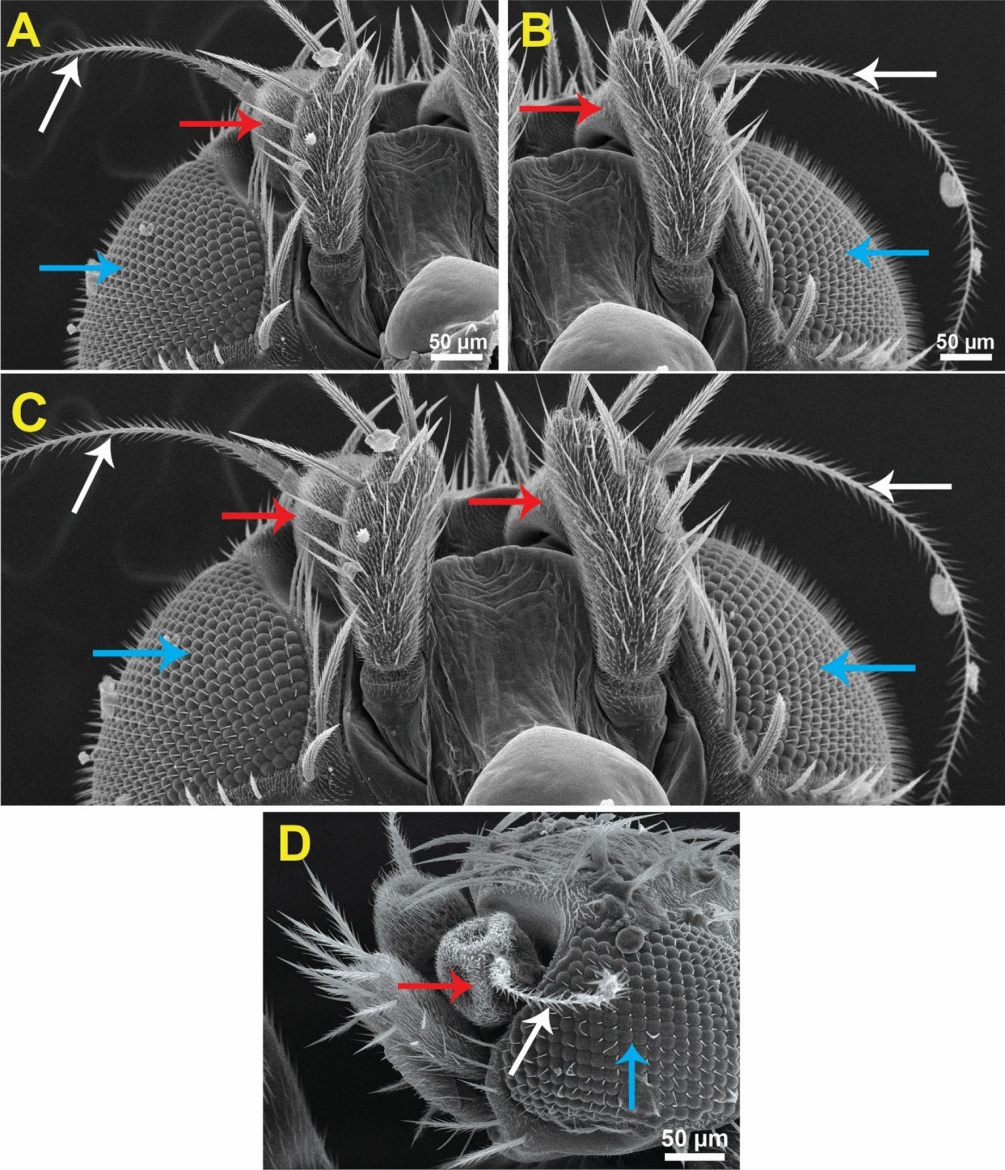
SEM micrographs of the *M. scalaris* head structure show the third antennal segment or the first flagellar segment (red arrows), the arista (white arrows), and the compound eyes (blue arrows). (**A**) and (**B**) Frontal views of the head structure, while (**C**) merged SEM micrographs for both (**A**) and (**B**) for better visualization of the whole head region of the fly. (**D**) Lateral view of the head region.

**Figure 7:**
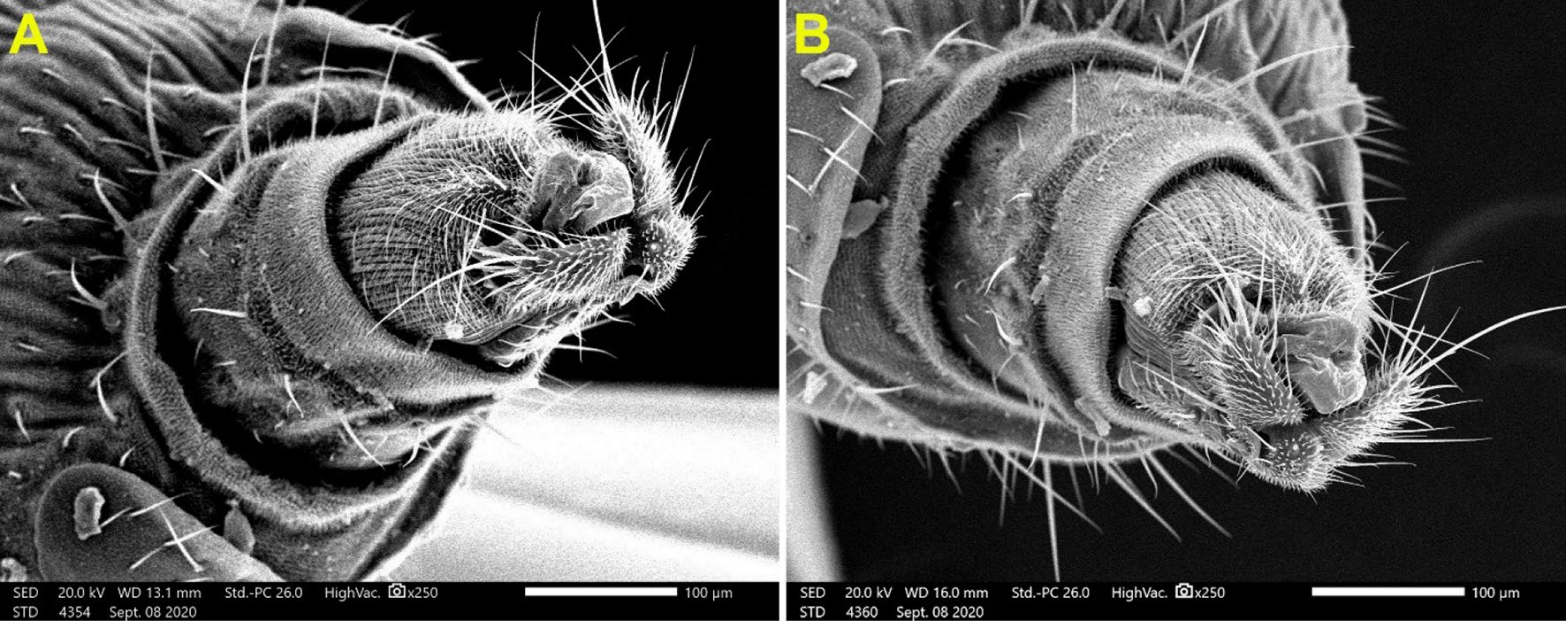
(**A**) and (**B**) SEM micrographs of female *M. scalaris* Illustrate ventral view of female ovipositor.

**Figure 8:**
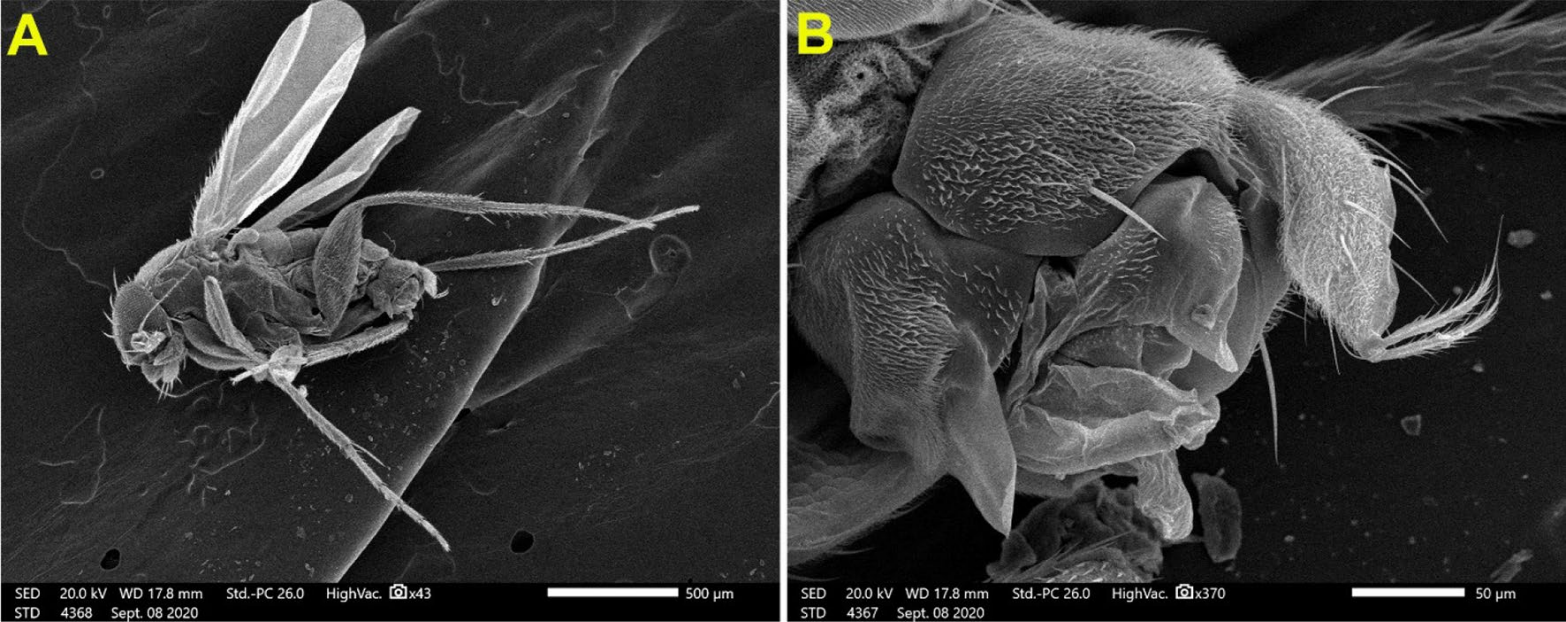
(**A**) SEM micrograph of male *M. scalaris* shows the whole bodies of the male scuttle flies and (**B**) SEM micrograph of male *M. scalaris* displays the apex of the male abdomen.

**Figure 9:**
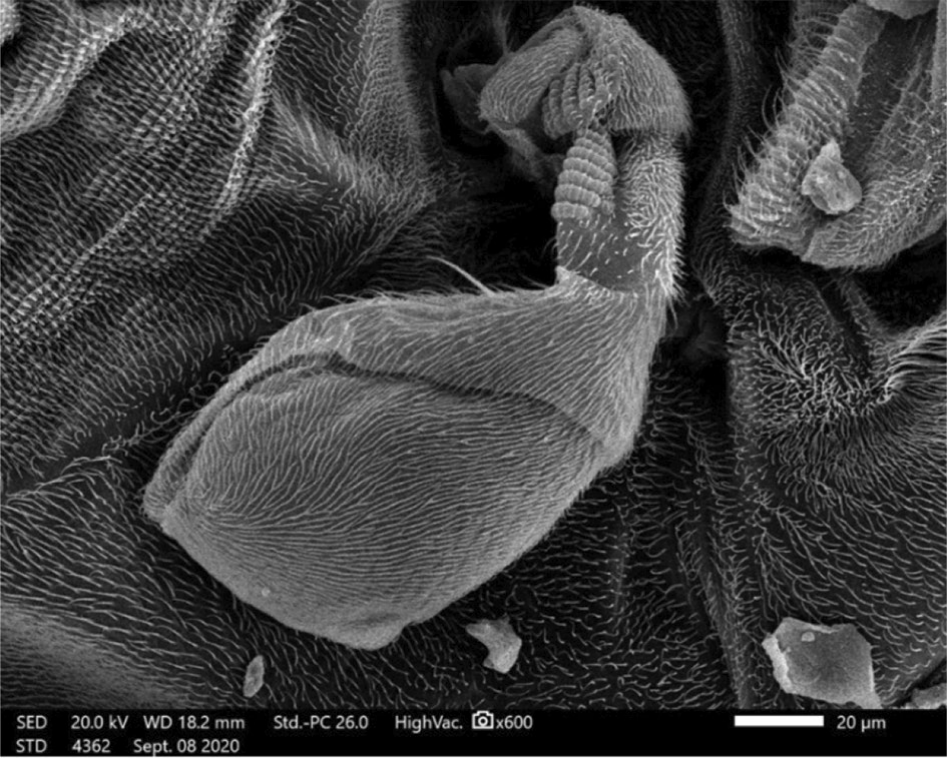
SEM micrograph of the *M. scalaris* shows haltere.

**Figure 10:**
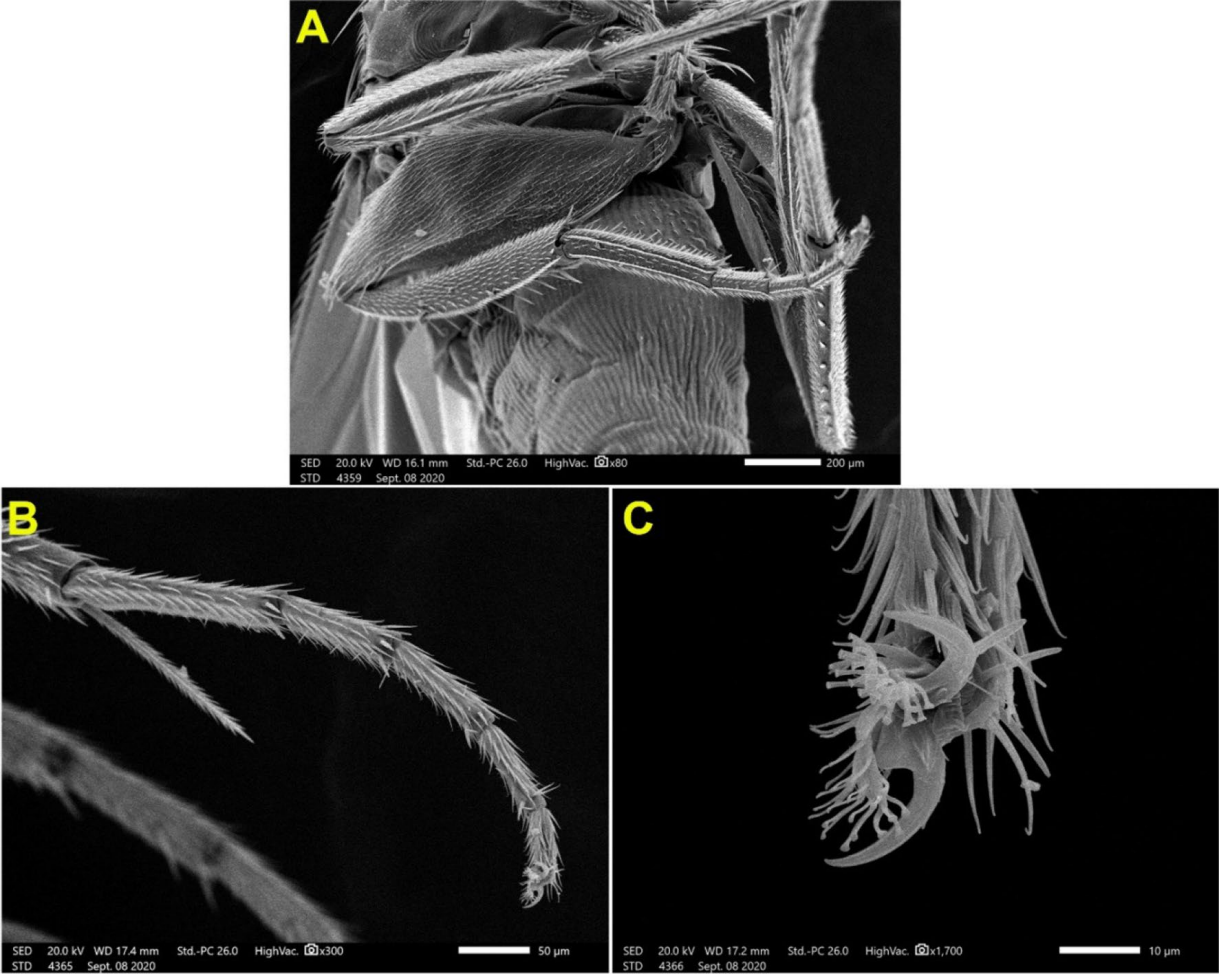
SEM micrographs of *M. scalaris* hind leg structure. (**A**) Hind femur, tibia, and tarsus. (**B**) and (**C**) SEM micrographs of *M. scalaris* hind tarsus, with enlarged pretarsal segment, respectively.

Medially at the tip of the labellum, a crescent-shaped membranous lobe could be perceived. Additionally, both the lateral and ventral surfaces of the labellum pear long and short trichoid sensilla, respectively, arise from a socket at their base. Moreover, Fig. 5 demonstrates the sexual dimorphism of male and female flies of the genus *Megaselia*, as the surface structure of the female labellum is exclusively smooth and lacks the microtrichia along with the presence of hooks in the labeler groove. Additionally, the antennal structure of these scuttle flies could be detectable as illustrated in Fig. 6. Figure 7 depicts the examination of the abdominal end of the female, revealing a noticeable ovipositor**.** Furthermore, a male scuttle fly was examined and the apex was noticed to have manifested external genitalia characteristic of phorid flies of the genus *Megaselia* as portrayed in Fig. 8. Other morphological structures, including halters, hind legs, tarsus, and pretarsal segments were examined and confirmed the species identification (Figs. 9 and 10).

### American cockroach behavior

The irregular cockroach’s behavior was observed due to *M. scalaris* sheltering. American cockroaches are commonly known for their active nature and tendency to exhibit fast-running and constant attempts to escape. However, this typical behavior commenced to alter with the emergence of an abnormal behavior pattern for roaches inside their cages. Zombification or zombie-like behavior could be defined as a leading observation of this abnormal behavior. Precisely, it could be perceived that American cockroaches negatively responded to the light, implying a significant alteration in their normal behavior. Besides, they materialized sick since they lost the normal fast movement and just retained slow walking. In addition to this zombie-like movement, we found that they have unfolded wings combined with no intention to fly. Furthermore, another characteristic observed in their morphology is the cutting of antennae and their emergence as short bristles. Moreover, decapitated roaches were found with the partially eaten head separated from the rest of the body. All these observations evidence the incidence of a parasitic species in our roaches.

**Figure 11:**
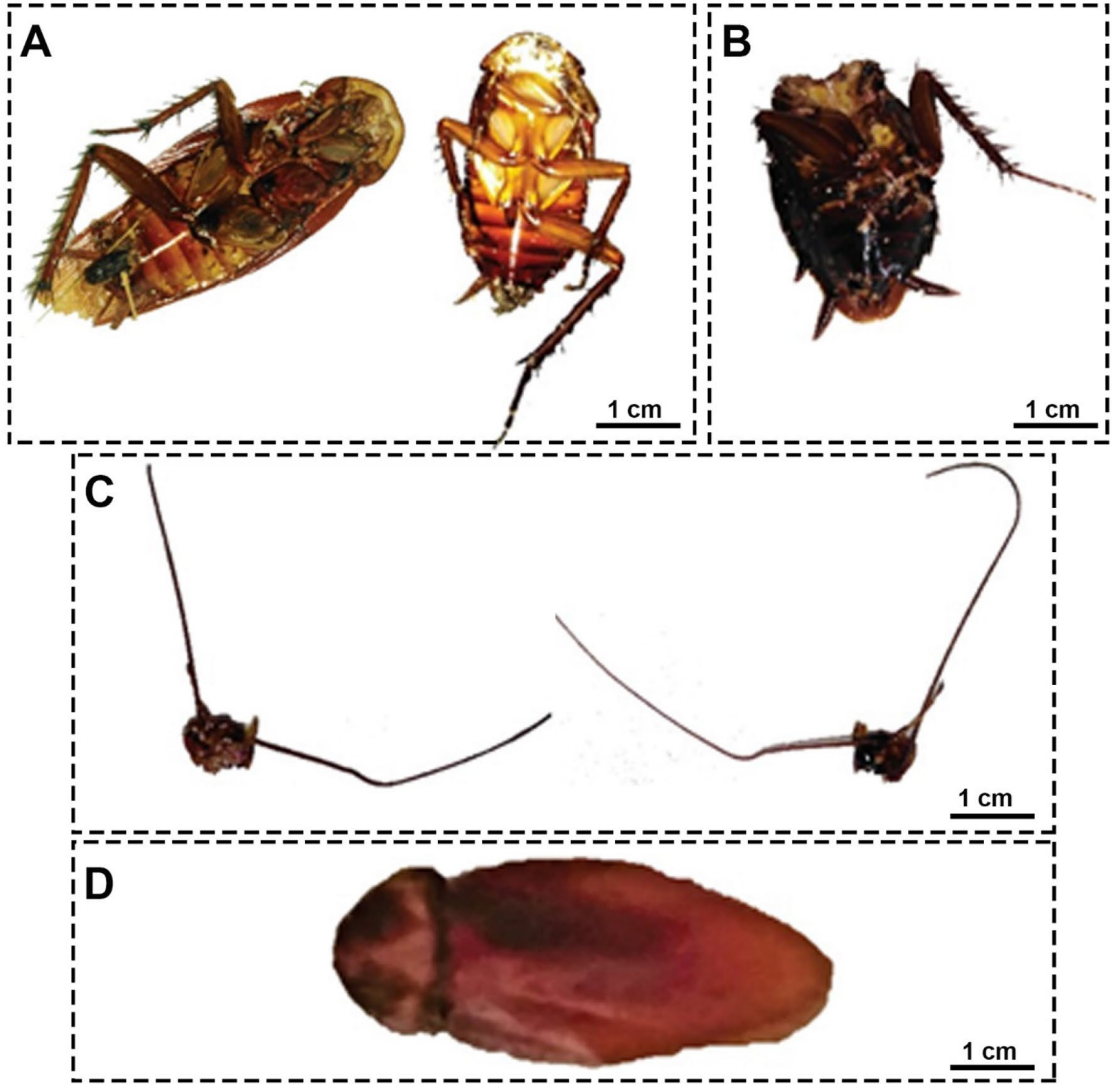
Several characteristics of *P. americana* were observed in this study as a consequence of infection with scuttle flies. (**A**) Decapitated *P. americana* with the entire body present. (**B**) *P. americana* with only the metathorax and abdomen present. (**C**) Separated head of the *P. americana*. (**D**) *P. americana* with only the pronotum and wings present.

After two days of the strange zombification behavior of the members of the nidus, the dead bodies of roaches were found. The attention-grabbing thing was the shape of dead cockroaches’ corpses. Some bodies were found without relatively much damage, but with a hole in the head region, while the neck was eaten. Other cockroaches were decapitated with mostly consumed heads and necks, and the rest of the body appeared intact. The third form has only the metathorax and abdomen present. The last shape but the least form present is that only the pronotum and forewings are present with bodies completely consumed as shown in Fig. 11.

## Discussion

In the current study, our attention was drawn to the peculiar and unexpected deaths of several American cockroaches in our cockroach culture, and all colonies died following a similar approach. This was the first noticeable observation, which drove us to understand and explain this phenomenon. Upon careful examination, it could be observed that their carcasses were entirely consumed by an unknown organism. Furthermore, several alterations in the cockroach's behavior were perceived, along with the presence of unknown tiny insects that exhibited erratic flight patterns within the cockroach colonies. The significance of these surveillances lies in the fact that such tiny parasitoids, measuring less than 3 mm in length^11^, are capable of parasitizing adult *P. americana* roaches, which measure between 34 and 53 mm in length^7^ in a tight sealing containers. This is inconsistent with Robinson^27^, who reported that eggs of *M. scalaris* are rarely oviposited in live cockroaches. Furthermore, he showed that oviposition was accomplished by the gravid female *M. scalaris* on organic remains. Additionally, he described its larval infestation of food and decaying roaches’ corpora in the rearing containers. However, no infestation could be perceived when American cockroaches fed on eggs of *M. scalaris*, which corroborated its parasitoidism in cockroaches. Although *M. scalaris* shows a preference for oviposition in dead cockroaches, including *P.* americana^28^, in our study all roaches were infested when they were alive.

These findings shed light on the exceptional adaptation of these small flies and their potential application as biological control agents in overwhelming roach populations. At first sight, it was challenging to identify these insects since they are not regular cockroach parasitoids. However, their aggressiveness and tenacity in attacking cockroaches indicated that they might have a role as aggressors. Upon sampling and morphological analysis, it was determined that these insects belong to the order Diptera, specifically to the family Phoridae. The Phoridae family of flies is characterized by its larval predatory behavior toward various organisms, including insects and small invertebrates^29,30^. As a result of the identification of Phoridae as aggressors toward American cockroaches, we could gain pivotal insights into the cause of death. Among Phoridae family, the flies infesting the American cockroaches were identified as *M. scalaris*, with distinct locomotory patterns, including ‘scuttle' in short bursts followed by rest periods^13^. Given that *M. scalaris* is polyphagous, a previous investigation reported its classification as necrophagous, sarcophagus, or saprophagous^31^.

The most evident observation is the decapitated adult American cockroaches, which can be attributed to the infestation of the parasitoid *M. scalaris*. Furthermore, this suggests that the insect targeted the cockroach's head to consume its contents. Moreover, this parasitoid insect utilizes cockroaches as a host to complete its life cycle, which involves four stages, including the egg, the larval, the pupal, and adult stages^18,30^. These findings are in line with those of Sánchez-Restrepo et al.^32^, who reported other dipteran phorid flies from the genus *Pseudacteon* (Diptera: Phoridae), demonstrating their ability to decapitate South American fire ants, including *Solenopsis invicta* and *Solenopsis richteri* (Hymenoptera: Formicidae). Additionally, El-Hawagry et al.^21^ showed *M. scalaris* parasitism on *N. viridula* with abnormal mortality recordings within the rearing cages. They reported on the consumption of the internal organs of these bugs and the impediment of their movement before their death because of their parasitoids. However, other phorids, including *Megaselia rufipes*, are facultative parasitoids of the honeybee *A. mellifera*, infesting only non-flying bees destined for death since parasitization could not be discernible in the normal honeybees^33^.

Interestingly, we observed remarkable behavioral alterations in the American cockroaches in the form of zombification behavior compared to healthy roaches. This implies the intricate interactions and relationships between the *M. scalaris* and the nervous system of the cockroaches. It is believed that any adaptive behavior could be attributed to connections between the environment and a living creature's brain and body; therefore, it is crucial to investigate the interactions between parasitoids and the host brain, body, and environment to comprehend the underlying mechanism of zombification behavior^34^. The term zombification has been previously postulated to describe the anomalous behavior of *P. americana* due to parasitic wasp infestations^35^. In a similar manner, a previous report exhibited a noticeable transformation in the typical aggressive behavior of soldier termites (*M. gilvus*) to non-aggressive with slow motion as a result of their parasitism by *M. scalaris*^20^. Furthermore, similar roving-like zombie behavior could be discernible in honey bees parasitized by the phorid *A. borealis*^19^. On the other hand, it has been shown that fungal parasites and viral genes can provoke zombie-like behaviors in their hosts, including insects^35^. Previous studies reported host manipulation in different taxa across the animal kingdom^35^. However, to the best of our knowledge, this is the first report to describe the phenotypic and behavioral manipulation of adult American cockroaches by their *M. scalaris* parasitoids. We report this observation of *M. scalaris* parasitism on healthy roaches without any kind of infection. This is in agreement with a previous investigation, which showed a similar infestation of *M. scalaris* in healthy bees^19^.

To delve into the morphological features of *M. scalaris*, we employed SEM analysis, which revealed the major characteristics of this fly. These comprehensive analyses were performed for the first time, providing valuable insights into the mouth parts and other structures of *M. scalaris* under magnification power. Besides, the adult’s sexual dimorphism was characterized through the examination of their external genitalia. However, it has been shown that the mouthparts of *M. scalaris* can also be used to distinguish between the sexes^13^. The host specificity and substantial impairment of *M. scalaris* to hosts make them potential candidates as a biological control agent, targeting specific pests. However, further research is required to comprehend the underlying mechanism of the behavioral alterations in the roaches. This can provide further insights into dynamic host-parasite interactions and intricate symbiotic relationships. On the other hand, a common consequence of adaptive host manipulation is fatal attraction, which was observed in several mammals toward their predators. Considering the parasitoid performance of *M. scalaris* on various insects^10^, larvae parasitized various vertebrates, including humans, eliciting both intestinal and wound myiasis^18^. Thus, this species has garnered medical importance owing to its critical role in relation to animal and human myiasis^10^. From Australia to Trinidad, numerous genuine cases of phorid larvae intestinal myiasis were reported^18^. Ingestion of *M. scalaris* eggs or larvae from uncooked or cold food usually results in infestation^18^. Therefore, birds or other cockroaches’ natural predators can be prone to intestinal myiasis. Moreover, in some cultures, entomophagy is a common feature of consuming various insects, including cockroaches^36^, making those populations more vulnerable to intestinal myiasis. However, the fate of *M. scalaris* eggs and larvae in the hostile conditions of the stomach has not been investigated^18^. Therefore, it is imperative to consider the shortcomings and potential disadvantages before using phorid flies as a biocontrol agent. In this context, further research and assessment are essential to define their efficacy and safety in different scenarios. It would be interesting to identify the enzymes produced by *M. scalaris* larvae to dominate and invade its hosts, which could elucidate its infestation mechanism and help devise novel approaches as a biocontrol agent, reducing the drawbacks to the environment and human health.

## Conclusions

To sum up, this study demonstrated the incidence of the scuttle fly, *M. scalaris*, infesting the American cockroach, *P. americana*. As a result of their endoparasitoids, the scuttle fly attacked the heads of cockroaches, resulting in zombification-like behavior. Moreover, we showed for the first time valuable details about the morphological properties of *M. scalaris* using SEM. Further studies are warranted before launching the application of this scuttle fly as a biological control agent to hinder the distribution of American cockroaches and their respective complications. However, our findings provide the first base for this potential application.

## Data Availability

The datasets used and/or analyzed during the current study are available from the corresponding author on reasonable request.

## References

[1] Li Q-J (2020). Mechanistic evaluation of gastro-protective effects of KangFuXinYe on indomethacin-induced gastric damage in rats. Chin. J. Nat. Med..

[2] Zeng C (2019). The role of *Periplaneta americana* (Blattodea: Blattidae) in modern versus traditional Chinese medicine. J. Med. Entomol..

[3] Liu J (2018). *Periplaneta americana* extract may attenuate renal fibrosis through inhibiting janus tyrosine kinase 2/signal transducer and activator of transcription 3 pathway. Pharmacology.

[4] Zhao Y, Yang A, Tu P, Hu Z (2017). Anti-tumor effects of the American cockroach, *Periplaneta*
*americana*. Chin. Med..

[5] Beutel RG, Friedrich F, Yang X-K, Ge S-Q (2014). Insect Morphology and Phylogeny.

[6] Nasirian H (2019). Contamination of cockroaches (Insecta: Blattaria) by medically important bacteriae: A systematic review and meta-analysis. J. Med. Entomol..

[7] Mullen GR, Durden LA (2009). Medical and Veterinary Entomology.

[8] Kruitwagen A, Beukeboom LW, Wertheim B (2018). Optimization of native biocontrol agents, with parasitoids of the invasive pest *Drosophila suzukii* as an example. Evolut. Appl..

[9] Kim H, Shin SE, Ko KS, Park SH (2020). The application of mitochondrial COI gene-based molecular identification of forensically important scuttle flies (Diptera: Phoridae) in Korea. BioMed. Res. Int..

[10] Ebrahimi L (2023). First record of Scuttle fly, *Megaselia* (M) *scalaris* (Loew) (Diptera: Phoridae) as a parasitoid of Sunn pest, *Eurygaster integriceps* Puton (Hemiptera: Scutelleridae) from Iran. Egypt. J. Biol. Pest Control.

[11] Durska E (2020). Preliminary data of the scuttle flies (Diptera: Phoridae) in the linden-oak-hornbeam forest of the Wigry National Park, North East Poland. Fragm. Faun..

[12] Zuha RM, Disney RHL (2020). Exploitation of cracked chicken eggs by scuttle flies (Diptera: Phoridae): The first record from Malaysia. Serangga.

[13] Pallavi J (2023). A complete morphological characterization of all life stages of the phorid fly *Megaselia scalaris*. Sci. Rep..

[14] Mongiardino Koch, N., Fontanarrosa, P., Padro, J. & Soto, I. M. First record of *Megaselia scalaris* (Loew)(Diptera: Phoridae) infesting laboratory stocks of mantids (*Parastagmatoptera tessellata*, Saussure) (2013).

[15] Feng D, Li J, Liu G (2020). The complete mitochondrial genomes of two scuttle flies, *Megaselia spiracularis* and *Dohrniphora cornuta* (Diptera: Phoridae). Mitochondrial DNA B.

[16] Zuha RM, Jenarthanan LX, Disney RH, Omar B (2015). Multiple species of scuttle flies (Diptera: Phoridae) as contaminants in forensic entomology laboratory insect colony. Trop. Biomed..

[17] Zhang X-S, Liu G-C, Zhang D-X, Shi C-M (2017). Novel trophic interaction: The scuttle fly *Megaselia scalaris* (Diptera: Phoridae) is a facultative parasitoid of the desert scorpion *Mesobuthus eupeus* mongolicus (Scorpiones: Buthidae). J. Nat. Hist..

[18] Disney RHL (2008). Natural history of the scuttle fly, *Megaselia scalaris*. Annu. Rev. Entomol..

[19] Ricchiuti L (2016). Infestation of *Apis mellifera* colonies by *Megaselia scalaris* (Loew, 1866) in Abruzzo and Molise regions, central-southern Italy. J. Apic. Res..

[20] Noknoy R, Sunantaraporn S, Phumee A, Siriyasatien P, Sanguansub S (2020). Parasitism of soldiers of the termite, *Macrotermes gilvus* (Hagen), by the scuttle fly, *Megaselia scalaris* (Loew) (Diptera: Phoridae). Insects.

[21] El-Hawagry MSA, Ebrahim AME, Nada MSE (2021). First detection of *Megaselia*
*scalaris* (Loew)(Diptera: Phoridae) as a facultative endoparasitoid of *Nezara*
*viridula* (L.)(Hemiptera: Pentatomidae). Egypt. J. Biol. Pest Control.

[22] Deshmukh SS (2021). First record of a parasitoid, *Megaselia* (M) *scalaris* (Diptera: Phoridae) of fall armyworm, *Spodoptera*
*frugiperda* (J. E. Smith) (Lepidoptera: Noctuidae) from India. Egypt. J. Biol. Pest Control.

[23] Tang Y (2021). First report on *Megaselia scalaris* Loew (Diptera: Phoridae) infestation of the invasive pest *Spodoptera frugiperda* smith (Lepidoptera: Noctuidae) in China. Insects.

[24] Arafat EA (2023). Entomotherapeutic role of *Periplaneta americana* extract in alleviating aluminum oxide nanoparticles-induced testicular oxidative impairment in migratory locusts (*Locusta migratoria*) as an ecotoxicological model. Antioxidants.

[25] Arafat EA, El-Samad LM, Moussian B, Hassan MA (2023). Insights into spermatogenesis in the migratory locust, *Locusta migratoria* (Linnaeus, 1758) (Orthoptera: Acrididae), following histological and ultrastructural features of the testis. Micron.

[26] Disney H (2012). Scuttle Flies: The Phoridae.

[27] Robinson W (1975). *Megaselia* (M.) *scalaris* (Diptera: Phoridae) associated with laboratory cockroach colonies. Proc. Entomol. Soc. Wash..

[28] Tsuda Y, Kamezaki H (2021). Preference of ovipositing *Megaselia scalaris* for dead adults of 6 different species of cockroach. Med. Entomol. Zool..

[29] Hsieh H-Y, Perfecto I (2012). Trait-mediated indirect effects of phorid flies on ants. Psyche.

[30] Namaki-Khameneh R, Khaghaninia SL, Disney RH, Maleki-Ravasan N (2021). The scuttle flies (Diptera: Phoridae) of Iran with the description of *Mahabadphora aesthesphora* as a new genus and species. PLoS One.

[31] Alam MS, Ahmed KA, Begum RA, Shahjahan RM (2016). Identification of *Megaselia scalaris* (Diptera: Phoridae) based on morphology and mitochondrial 16S rRNA and COI gene sequences. Dhaka Univ. J. Biol. Sci..

[32] Sánchez-Restrepo AF (2020). A Species delimitation approach to uncover cryptic species in the South American fire ant decapitating flies (Diptera: Phoridae: *Pseudacteon*). PLoS One.

[33] Dutto M, Ferrazzi P (2014). *Megaselia rufipes* (Diptera: Phoridae): A new cause of facultative parasitoidism in *Apis mellifera*. J. Apic. Res..

[34] Osuka K (2019). Zombification of insects as a model for searching the source of various behaviors of living organisms. J. Robot. Mechatron..

[35] Libersat F, Delago A, Gal R (2009). Manipulation of host behavior by parasitic insects and insect parasites. Annu. Rev. Entomol..

[36] Liceaga AM (2022). Advances in Food and Nutrition Research.

